# Peripheral Nerve Involvement in Friedreich's Ataxia Characterized by Quantitative Magnetic Resonance Neurography

**DOI:** 10.1111/ene.70121

**Published:** 2025-03-25

**Authors:** Heike Jacobi, Markus Weiler, Georges Sam, Sabine Heiland, John M. Hayes, Martin Bendszus, Wolfgang Wick, Jennifer C. Hayes

**Affiliations:** ^1^ Department of Neurology Heidelberg University Hospital Heidelberg Germany; ^2^ Department of Neuroradiology Heidelberg University Hospital Heidelberg Germany; ^3^ Division of Experimental Radiology, Department of Neuroradiology Heidelberg University Hospital Heidelberg Germany; ^4^ Department of Neurology University of Michigan Ann Arbor USA; ^5^ Clinical Cooperation Unit Neurooncology German Cancer Research Center/DKTK Heidelberg Germany; ^6^ Department of Radiology University of Michigan Ann Arbor USA

**Keywords:** electrophysiology, Friedreichs ataxia, magnetic resonance neurography (MRN), magnetization transfer contrast imaging, T2 relaxometry

## Abstract

**Background:**

Friedreich's ataxia (FRDA) affects both the central and peripheral nervous system. Peripheral nerve involvement manifests predominantly as a progressive sensory neuropathy caused by dorsal root ganglionopathy. An additional direct involvement of peripheral nerves leading to abnormal myelination is increasingly discussed. Here, we characterize lower extremity peripheral nerve involvement in FRDA by quantitative magnetic resonance neurography (MRN).

**Methods:**

Sixteen genetically confirmed FRDA patients and 16 age‐/sex‐matched controls were prospectively enrolled. Patients underwent neurologic examinations and nerve conduction studies (NCS). Large‐coverage MRN of sciatic and tibial nerves was conducted utilizing dual‐echo turbo‐spin‐echo sequences with spectral fat saturation for T2‐relaxometry, and two gradient‐echo sequences with and without off‐resonance saturation rapid frequency pulses for magnetization transfer contrast imaging. Microstructural and morphometric MRN markers including T2‐relaxation time (T2_app_), proton spin density (*ρ*), magnetization transfer ratio (MTR), and cross‐sectional area (CSA) were calculated to characterize nerve lesions.

**Results:**

Tibial nerve *ρ* and T2_app_ were markedly decreased in FRDA at the thigh (*ρ*: 368.4 ± 11.0 a.u.; T2_app_: 59.5 ± 1.8 ms) and lower leg (*ρ*: 337.3 ± 12.6 a.u.; T2_app_: 53.9 ± 1.4 ms) versus controls (thigh, *ρ*: 458.9 ± 9.5 a.u., *p* < 0.0001; T2_app_: 66.3 ± 0.8 ms, *p* = 0.0019; lower leg, *ρ*: 449.9 ± 12.1 a.u., *p* < 0.0001; T2_app_: 62.4 ± 1.2 ms, *p* < 0.0001) and correlated well with clinical scores, disease duration, and NCS. MTR and CSA did not differentiate between FRDA and controls.

**Conclusion:**

Our study results provide a profound characterization of peripheral nerve involvement in FRDA. The identified good correlation between *ρ* and T2_app_ with clinical symptom scores and NCS suggests that parameters of T2 relaxometry may become relevant biomarkers to monitor disease progression and therapeutic responses in potential future clinical trials.

## 1 Introduction

Friedreich's ataxia (FRDA) is one of the most common autosomal recessive genetic ataxias. It is caused by biallelic pathogenic alterations in the frataxin gene on chromosome 9q13, usually caused by an intronic guanine‐adenine‐adenine (GAA) trinucleotide repeat expansion [1]. The age of disease onset inversely correlates with the number of minor GAA repeats [2].

FRDA usually begins in childhood or early adulthood [3] and is a multisystemic disorder affecting both the central (CNS) and peripheral nervous system (PNS), often accompanied by cardiomyopathy, skeletal abnormalities (scoliosis, pes cavus) and diabetes [4]. The phenotype is usually characterized by progressive unsteadiness of stance and gait, followed by slurred speech, eye movement disorders, areflexia, leg weakness, and loss of sensation [4]. Although the phenotype is very complex and variable, peripheral sensory loss is a consistent finding that contributes significantly to stance and gait ataxia. Dorsal root ganglionopathy, leading to progressive sensory neuronopathy, particularly of large fibers, is one of the main hallmarks of PNS involvement [5, 6]. Evidence from neuropathologic and sonographic studies points towards additional direct involvement of peripheral nerves manifesting in abnormal myelination [7].

In this prospective study, we characterize peripheral nerve involvement in FRDA clinically, electrophysiologically, and by application of quantitative magnetic resonance neurography (MRN) in comparison to healthy controls. MRN is an emerging technique that has previously localized and quantified peripheral nerve involvement in vivo in various diffuse neuropathies [8, 9, 10, 11, 12, 13]. Nerve lesions in FRDA were quantified by T2 relaxometry, magnetization transfer contrast (MTC) imaging, and morphometric quantification.

## 2 Methods

### 2.1 Standard Protocol Approvals, Registration, and Patient Consents

This prospective case–control study was approved by the institutional ethics board (University of Heidelberg; S‐398/2012), and written informed consent was obtained from all participants according to the Declaration of Helsinki.

### 2.2 Study Design, Neurologic and Electrophysiologic Assessments

We enrolled 16 patients with a genetically confirmed diagnosis of FRDA (6 males, 10 females, mean age 37.2 ± 3.3 years, range 19–64) between August 2019 and June 2024. We also recruited 16 age‐ and sex‐matched healthy volunteers (6 males, 10 females, mean age 37.2 ± 3.5 years, range 19–64 years). Exclusion criteria for all groups were age < 18 years, pregnancy, any contraindications for MRI, prior treatments with potentially neurotoxic agents, concomitant causes for the development of a polyneuropathy (PNP) such as diabetes mellitus, severe hypothyroidism, or Vitamin B12 deficiency, as well as any malignant or infectious diseases or neurological disorders other than FRDA.

As the clinical phenotype can vary depending on the age of disease onset, we further divided our patients into the following subgroups for additional analyses: FRDA patients with a (i) typical (young) age of onset < 25 years (FRDAtyp), (ii) late onset > 25 years (LOFA), and (iii) very‐late‐onset > 40 years (VLOFA). Due to the small group sizes in the LOFA (*n* = 2) and VLOFA groups (*n* = 2), we only report results for all FRDA patients and additionally for FRDAtyp patients.

A detailed medical history was taken from all FRDA patients and controls. Comprehensive neurologic examinations were performed in the FRDA group including documentation of the Scale for Assessment and Rating of Ataxia (SARA; H.J.). Nerve conduction studies (NCS) assessed distal motor latencies (DML), compound muscle action potentials (CMAP), and nerve conduction velocities (NCV) of the peroneal and tibial nerves. Sensory nerve action potentials (SNAP) and NCVs were measured for the sural nerve (G.S.; M.W.). Skin temperature was controlled at a minimum of 32°C.

Determination of existing polyneuropathy was solely based on electrophysiologic results without accounting for clinical presentations and neurologic examination results to reduce the risk of potentially overlapping clinical symptoms derived from degeneration of posterior columns and the corticospinal tract being viewed as PNP symptoms.

### 2.3 MRN Protocol

All participants were examined feet first, in a supine position in a 3.0 Tesla MR scanner (Magnetom PRISMA, Siemens‐Healthineers, Erlangen, Germany). A 15‐channel transmit‐receive extremity coil (INVIVO, Gainesville, FL, USA) was used for all imaging, and the following high‐resolution MRN protocol for T2 relaxometry and MTC imaging was carried out:

- T2‐relaxometry: Axial dual‐echo turbo‐spin‐echo 2D‐sequence with spectral fat saturation with four continuous imaging slabs covering the left leg: Slab 1: proximal thigh to mid‐thigh; slab 2: mid‐thigh to distal thigh with alignment of the distal edge of this imaging slab on the tibiofemoral joint space; slab 3: lower leg with alignment of its proximal edge with the tibiofemoral joint space; slab 4: ankle level with alignment of the distal edge on the tibiotalar joint space. One additional slab was acquired at the right leg, covering the region from the mid‐thigh to the distal thigh, with the distal edge of the imaging slab aligned to the tibiofemoral joint space. Sequence parameters were: Repetition time (TR) 5860 ms, lower echo time (TE_1_) 14 ms, higher echo time (TE_2_) 86 ms, field‐of‐view 170 × 170 mm^2^, matrix size 512 × 512, slice thickness 3.5 mm, interslice gap 0.35 mm, voxel size 0.3 × 0.3 × 3.5 mm^3^, flip angle 180°, 35 slices, acquisition time per slab 8:25 min.
- MTC imaging: Two axial three‐dimensional, gradient echo sequences with and without an off‐resonance saturation pulse (Gaussian envelop, duration 9984 μs, frequency offset 1200 Hz) were performed at the exact same slice position at the right mid‐ to distal thigh using the following identical sequence parameters: TR 50 ms, TE 4.92 ms, field‐of‐view 160 × 160 mm^2^, matrix size 256 × 256, band width 370 Hz/Px, slice thickness 3.5 mm, voxel size 0.6 × 0.6 × 3.5 mm^3^, flip angle 7°, 16 slices, acquisition time per sequence 3:48 min.

The total acquisition time, including survey scans, patient and coil repositioning, was approximately 75 min per participant.

### 2.4 T2 Relaxometry

After pseudonymization, all images were analyzed in ImageJ (version 1.52v; National Institutes of Health, Bethesda, MD, USA) by one neuroradiologist blinded to clinical data (J.C.H.). The circumference of the left tibial nerve and respective tibial fascicles within the sciatic nerve were manually delineated as the intraneural region of interest (ROI) on axial slices generated by the T2‐relaxometry sequence from the left proximal thigh down to the left tibiotalar joint and from the right mid‐ to distal thigh. To avoid artifacts due to signal inhomogeneities, we evaluated only the central 20 slices of each imaging slab, totaling 100 segmented slices per participant (80 slices on the left and 20 slices on the right).

Subsequently, the apparent T2 relaxation time (T2_app_, equation 1) and proton spin density (*ρ*, equation 2) were calculated by using data from the T2‐relaxometry sequences with SI = signal, TE_1_ = 14 ms, and TE_2_ = 86 ms [14, 15].

(1) $$T2\mathrm{app}=\frac{{\mathrm{TE}}_{2}-{\mathrm{TE}}_{1}}{\ln\left(\frac{\mathrm{SI}\left(\mathrm{TE}1\right)}{\mathrm{SI}\left(\mathrm{TE}2\right)}\right)}$$

(2) $$\rho =\frac{\mathrm{SI}\left({\mathrm{TE}}_{1}\right)}{\exp\left(\frac{{\mathrm{TE}}_{1}}{T2_{\mathrm{app}}}\right)}$$

After calculating mean values of tibial nerve *ρ* and T2_app_ per slice position for each participant, mean *ρ* and T2_app_ values were averaged for the thigh (imaging slabs 1 and 2) and the lower leg (imaging slabs 3 and 4).

### 2.5 Magnetization Transfer Contrast Imaging

The sciatic nerve circumference was manually delineated as intraneural ROI approximately 1 cm proximal to the nerve bifurcation on ten consecutive central axial slices generated by MTC imaging at the right mid‐ to distal thigh. All ROIs were primarily defined on images acquired by the gradient echo sequence without the off‐resonance saturation pulse and then subsequently transferred to the exact same position on the corresponding slices generated by the sequence with the off‐resonance saturation pulse by using the “synchronize windows” tool in ImageJ. The magnetization transfer ratio (MTR) was calculated according to the following equation, in which S_0_ is the signal without and S_1_ with the off‐resonance saturation pulse:

$$\mathrm{MTR}=100\times \frac{\left(S_{0-}S_{1}\right)}{S_{0}}$$

After calculating mean values of sciatic nerve MTR per slice position for each participant, mean MTR values were subsequently extracted and averaged over the ten analyzed slice positions in each participant.

### 2.6 Morphometric Quantification

The cross‐sectional area (CSA) of the tibial nerve and respective tibial fascicles within the sciatic nerve was evaluated by using the exact same ROIs on the same 20 central slices that were segmented for T2 relaxometry.

### 2.7 Statistical Analysis

Statistical analyses were performed in GraphPad Prism (Version 10.2.3; GraphPad Software Inc., La Jolla, CA, USA). Firstly, the normal distribution of each evaluated quantitative dataset was confirmed by using the Kolmogorov–Smirnov test. Differences in the evaluated MRN parameters between FRDA patients and healthy controls, of the thigh and the lower leg, and the left and right distal thigh were evaluated by an unpaired t‐test with Welch's correction. Correlation analyzes between MRN parameters and demographic (patient age, sex, height, weight, body mass index), clinical (duration of symptoms, SARA, pallaesthesia) and electrophysiologic (tibial and peroneal NCV, DML, and CMAP, sural nerve NCV and SNAP) results were performed by calculating Pearson's correlation coefficients. Statistical tests were two‐tailed, and *p*‐values ≤ 0.05 were regarded as statistically significant. Results are documented as mean values ± standard error of the mean.

### 2.8 Data Availability Statement

All data used to conduct this study are documented in the Section 2. Additional anonymized datasets will be shared at the request of any qualified investigator.

## 3 Results

### 3.1 Clinical and Electrophysiologic Data

FRDA patients and healthy controls did not differ in important demographic variables such as age (FRDA: mean age 37.2 ± 3.3 years; controls: 37.2 ± 3.5 years; *p* = 0.99), sex (FRDA: 6 males, 10 females; controls: 6 males, 10 females), body weight (FRDA: 72.2 ± 3.0 kg; controls: 74.4 ± 4.1 kg; *p* = 0.66), body height (FRDA: 170 ± 2 cm; controls: 174 ± 2 cm; *p* = 0.29), or BMI (FRDA: 24.8 ± 0.8 kg/m^2^; controls: 24.6 ± 1.2 kg/m^2^; *p* = 0.89). Most FRDA patients showed a mild axonal, solely sensory PNP. Detailed clinical and electrophysiologic data of FRDA patients are given in Table 1.

**TABLE 1:** Clinical and electrophysiologic data.

Parameter	FRDA (*n* = 16)
SARA (0–40 points)	21.5 ± 2.2
Disease duration (years)	15.3 ± 2.1
Mobility (independent/walking aids/wheelchair)	3/3/10
PTR areflexia/normal/hyperreflexia	13/3/0
ATR areflexia/normal/hyperreflexia	14/2/0
Pallhypesthesia (none/mild/moderate/severe)	0/1/12/4
SNAP (μV)	
RSN	3.6 ± 1.0
LSN	4.6 ± 1.1
CMAP (mV)	
RPN	6.2 ± 0.7
LPN	5.9 ± 0.7
RTN	19.0 ± 1.9
LTN	18.2 ± 1.9
DML (ms)	
RPN	4.7 ± 0.4
LPN	4.6 ± 0.2
RTN	4.1 ± 0.1
LTN	4.0 ± 0.1
NCV (m/s)	
RPN	43.6 ± 1.4
LPN	43.9 ± 1.1
RTN	46.1 ± 1.3
LTN	45.0 ± 1.2
RSN	47.2 ± 1.1
LSN	46.4 ± 0.9

Abbreviations: ATR, Achilles tendon reflex; CMAP, compound muscle action potential; DML, distal motor latency; FRDA, Friedreich's ataxia; LPN, left peroneal nerve; LSN, left sural nerve; LTN, left tibial nerve; NCV, nerve conduction velocity; Pallhypesthesia, none = 8/8, mild = > 5/8, moderate = 2–5/8, severe = < 2/8; PTR, patellar tendon reflex; RPN, right peroneal nerve; RSN, right sural nerve; RTN, right tibial nerve; SARA, scale for assessment and rating of ataxia; SNAP, sensory nerve action potential.

### 3.2 MRN Data

#### 3.2.1 Proton Spin Density (*ρ*)

**FIGURE 1:**
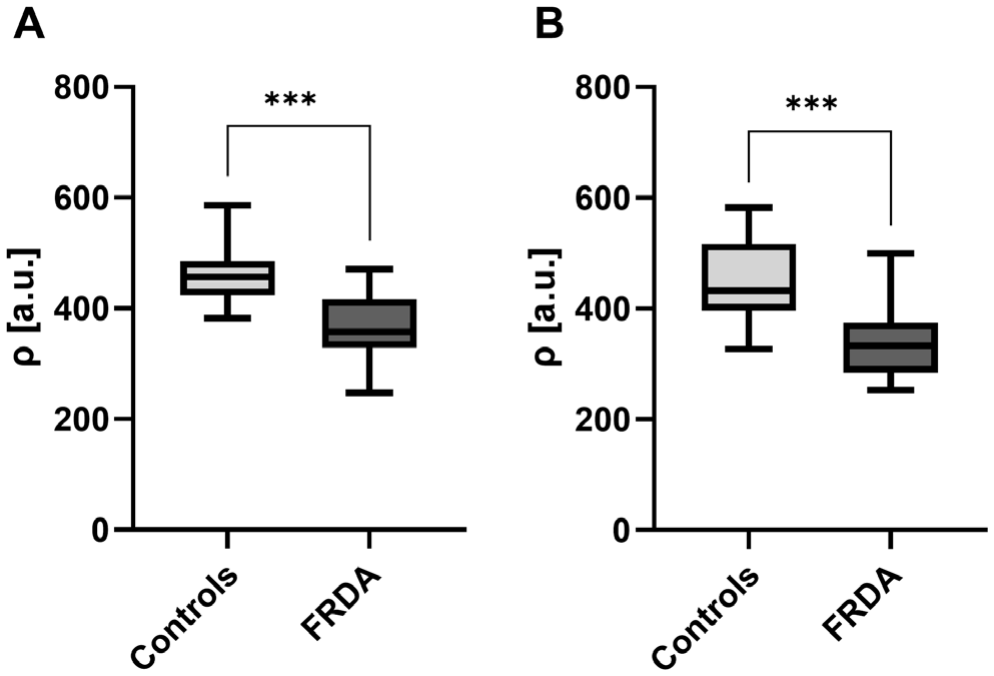
Proton spin density. Mean values of tibial nerve *ρ* were plotted separately for healthy controls and FRDA patients at the thigh (A) and at the lower leg (B) in a box and whisker plot. Tibial nerve *ρ* was markedly lower in FRDA patients compared to controls at the thigh (A) and at the lower leg (B). *ρ* = proton spin density; FRDA = Friedreich's ataxia. Significant differences are indicated by either * = significant (*p* ≤ 0.05), or *** = highly significant (*p* ≤ 0.001).

**FIGURE 2:**
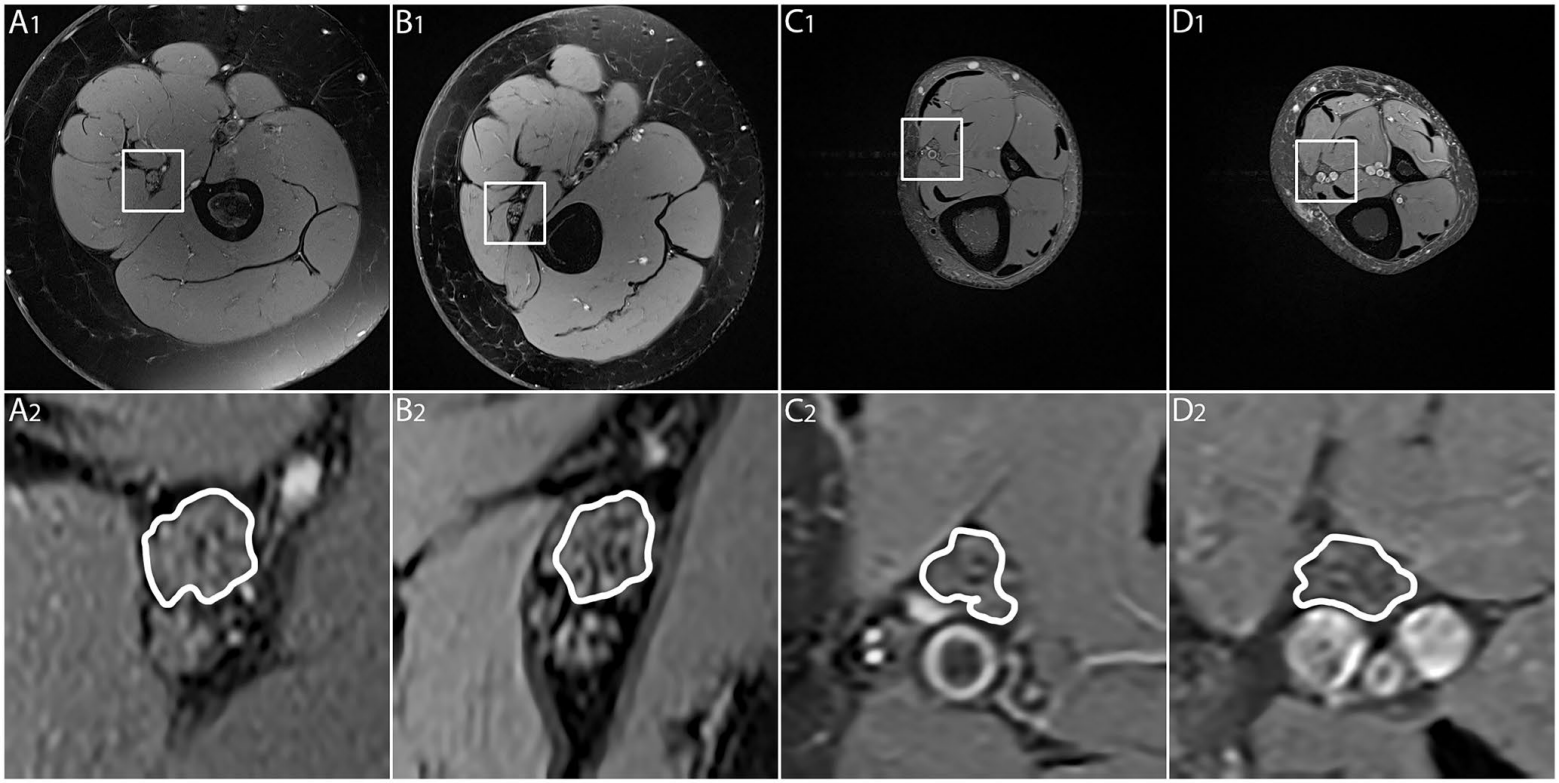
MRN source images. Representative magnetic resonance neurography (MRN) images (axial dual‐echo turbo spin echo relaxometry sequences with spectral fat saturation) at the left midthigh (A_1_—B_2_) and at the lower leg (C_1_—D_2_) are shown at equal slice positions and equal magnification in a representative healthy control (A_1/2_ and C_1/2_) and a representative FRDA patient (B_1/2_ and D_1/2_). A_1_, B_1_, C_1_, and D_1_ present complete cross‐sectional images of the respective thigh or lower leg, while the details below show the tibial fascicles within the sciatic nerve (A_2_, B_2_) and the tibial nerve (C_2_, D_2_) encircled in white. Note that only a minimal decrease in T2w signal was observed in the representative FRDA patient compared to the healthy control at the thigh and lower leg, resulting from the observed combined decrease in *ρ* and T2_app_ in FRDA. No relevant change in tibial nerve CSA was noticed in FRDA patient compared to the healthy control at either location.

There was a marked decrease in tibial nerve *ρ* in FRDA patients at the thigh (368.4 ± 11.0 a.u.) and at the lower leg (337.3 ± 12.6 a.u.) compared to controls (thigh: 458.9 ± 9.5 a.u., *p* < 0.0001; lower leg: 449.9 ± 12.1 a.u., *p* < 0.0001; Figures 1 and 2). When analyzing only FRDAtyp patients, tibial nerve *ρ* was also substantially lower at the thigh (368.9 ± 14.1 a.u.; *p* < 0.0001) and lower leg (333.6 ± 15.6 a.u.; p < 0.0001) compared to controls.

A proximal‐to‐distal gradient in tibial nerve *ρ* from the thigh to the lower leg was not observed in FRDA (*p* = 0.07) or controls (*p* = 0.56). Differences between the left and right mid‐ to distal thigh did not exist in FRDA (0.36) or controls (*p* = 0.58).

No correlations between tibial nerve *ρ* at the thigh or lower leg with any demographic parameter were detected in the group of healthy controls. In FRDA patients, tibial nerve *ρ* at the thigh inversely correlated with the SARA score (r = −05374, *p* = 0.0046) and disease duration (r = −0.6307, *p* = 0.0006), while positive correlations were detected between tibial nerve *ρ* and sural nerve SNAP (right: *r* = 0.5951, *p* = 0.0248; left: *r* = 0.5373, *p* = 0.0475). At the lower leg, tibial *ρ* inversely correlated with the SARA score (−0.5029, *p* = 0.0104) and disease duration (r = −0.6662, *p* = 0.0003). Further consistent correlations between tibial nerve *ρ* at thigh or lower leg level and any of the remaining demographic, clinical, or electrophysiologic parameters did not exist in FRDA patients.

#### 3.2.2 Apparent T_2_ ‐Relaxation Time (T2_app_)

**FIGURE 3:**
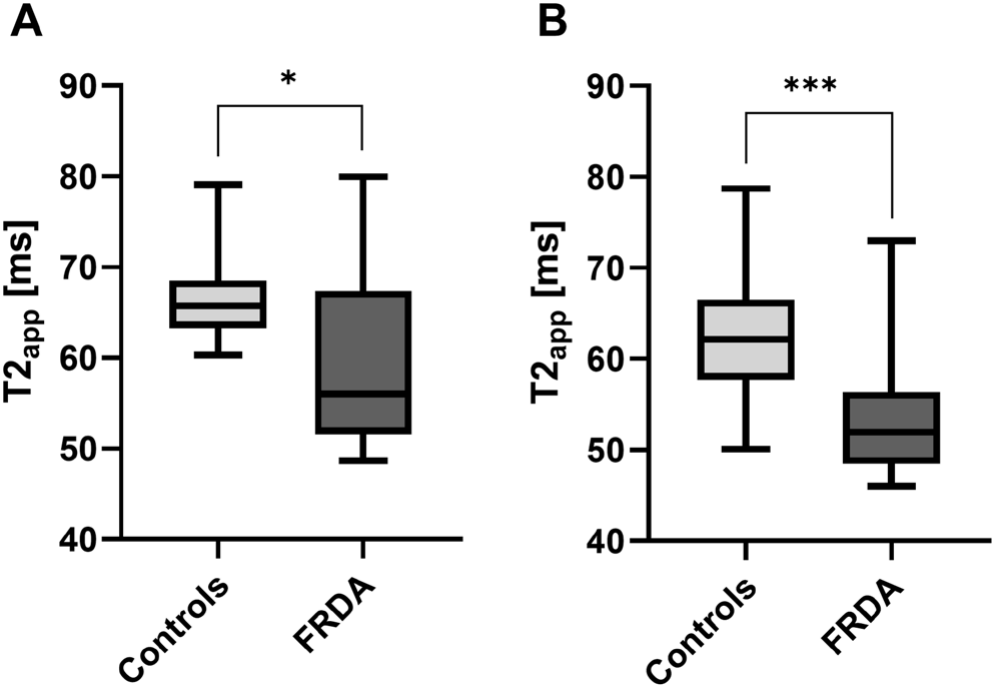
T2‐relaxation time. Mean values of tibial nerve T2_app_ were plotted separately for healthy controls and FRDA patients at the thigh (A) and lower leg (B) in a box and whisker plot. T2_app_ differentiated well between healthy controls and FRDA patients by being markedly decreased in FRDA patients at the thigh and lower leg compared to healthy controls. T2_app_ = T2‐relaxation time; FRDA = Friedreich's ataxia. Significant differences are indicated by either * = significant (*p* ≤ 0.05), or *** = highly significant (*p* ≤ 0.001).

Tibial nerve T2_app_ at thigh level was markedly lower in FRDA patients (59.5 ± 1.8 ms) than in controls (66.3 ± 0.8 ms; *p* = 0.0019; Figures 2 and 3a) and was also lower at lower leg level in FRDA patients (53.9 ± 1.4 ms) than in controls (62.4 ± 1.2 ms; *p* < 0.0001; Figures 2 and 3b). In FRDAtyp patients, tibial nerve T2_app_ was also lower at the thigh (59.2 ± 2.4 ms; *p* = 0.0091) and at the lower leg (52.5 ± 1.4 ms; *p* < 0.0001) compared to controls.

A proximal‐to‐distal gradient in tibial nerve T2_app_ from the thigh to the lower leg was observed in FRDA (*p* = 0.0184) and in controls (*p* = 0.0432). Tibial nerve T2_app_ did not differ between the left and right mid‐ to distal thigh in FRDA patients (*p* = 0.47) and controls (*p* = 0.62).

We found no correlations between tibial nerve T2_app_ at the thigh or lower leg with any demographic parameters in the group of healthy controls.

In FRDA patients, tibial nerve T2_app_ at the thigh inversely correlated with the SARA score (*r* = −0.3900, *p* = 0.0443) and disease duration (*r* = −0.7892, *p* < 0.0001), while positively correlating with tibial nerve CMAP (right: *r* = 0.6391, *p* = 0.0003; left: *r* = 0.5417, *p* = 0.0035) and sural nerve SNAP (right: *r* = 0.8655, *p* < 0.0001; left: *r* = 0.6473, *p* = 0.0091). Tibial nerve T2_app_ at the lower leg correlated inversely with the SARA score (*r* = −0.5357, *p* = 0.0058) and disease duration (*r* = −0.6634, *p* = 0.0003). We did not detect any further consistent correlations between tibial nerve *ρ* at the thigh or lower leg with any other demographic, clinical, or electrophysiologic parameters in FRDA.

#### 3.2.3 Magnetization Transfer Ratio (MTR)

Sciatic nerve MTR at the right mid‐ to distal thigh did not differentiate between FRDA patients (31.6% ± 0.7%) and healthy controls (32.0% ± 0.8%; *p* = 0.76). When evaluating only those FRDA patients who manifested at a typical age of onset, we again did not observe a difference in sciatic nerve MTR in FRDAtyp patients (31.2% ± 0.8%) versus controls (*p* = 0.48). Therefore, we did not perform any correlation analyses.

#### 3.2.4 Cross Sectional Area

Tibial nerve CSA did not differ between FRDA patients and healthy controls at the thigh (FRDA: 14.3 ± 0.9 mm^2^; controls: 13.5 ± 0.3 mm^2^; *p* = 0.39) and at the lower leg (FRDA: 7.3 ± 0.3 mm^2^; controls: 7.0 ± 0.2 mm^2^; *p* = 0.53). Differences in tibial nerve CSA were also not observed in FRDAtyp patients (thigh: 15.2 ± 1.1 mm^2^; lower leg: 7.3 ± 0.4 mm^2^) versus controls at the thigh (*p* = 0.16) or lower leg (*p* = 0.48).

Consistent with the physiologic decrease in nerve caliber from proximal to distal, tibial nerve CSA was markedly higher at the thigh than at the lower leg in FRDA and controls (*p* < 0.0001, respectively). Side differences between CSAs of the right and left tibial nerve at mid‐ to distal thigh level were not observed in FRDA (*p* = 0.55) and controls (*p* = 0.47). As no differences in tibial nerve CSA were observed between FRDA patients and controls for either anatomic location, we did not perform any correlation analyses.

## 4 Discussion

Friedreich's ataxia is a multisystemic disorder affecting both the CNS and PNS. One of the main manifestations of PNS involvement is a dorsal root ganglionopathy leading to progressive sensory neuronopathy [5]. Increasing evidence for an additional direct involvement of peripheral nerves arose from a recent sonographic study [7]. However, the exact underlying pathomechanism and associated microstructural processes in the peripheral nerves are still not fully understood.

In this prospective study, we present a comprehensive characterization of lower extremity peripheral nerve involvement in FRDA using quantitative MRN in correlation with clinical and NCS examination findings and in comparison to healthy controls. Our results show that the quantitative MRN parameters tibial nerve *ρ* and T2_app_ are markedly decreased in FRDA compared to controls, while correlating well with the duration of symptoms, SARA score, and NCS parameters. In contrast, MTR and CSA failed to differentiate between FRDA and controls and can be disregarded as imaging biomarkers in FRDA.

The quantitative T2 relaxometry parameters *ρ* and T2_app_ serve as microstructural markers of nerve tissue integrity, reflecting the macromolecular composition and biochemical characteristics of nerve tissue in vivo [14, 16, 17]. Both markers have recently contributed substantially to the quantification of nerve lesions and subsequently improved the characterization and classification of a wide variety of PNPs and other diffuse neuropathies. This has positioned T2 relaxometry as an increasingly important technique in the development of new imaging biomarkers [9, 11, 12, 18, 19, 20, 21, 22].

It has been hypothesized that an increase in macromolecules in peripheral nerve tissue related to protein deposits or inflammatory reactions leads to an increase in *ρ*, for example caused by an accumulation of proteins in hereditary or systemic amyloidosis [11, 15, 19, 20], or by an accumulation of glycosylated end‐products in diabetes [9, 12]. On the contrary, a decrease in *ρ* has been associated with demyelination or axonal loss as it occurs, for example, in 5q‐linked spinal muscular atrophy secondary to a decay of lower motor neurons [10]. While the alterations in *ρ* seemingly reflect the concentration of hydrogen protons in a given volume, T2_app_ delivers information on the amount of free water molecules in nerve tissue [23].

The observed combined decrease in tibial nerve *ρ* and T2_app_ in our FRDA patients is similar to previous studies in other neurodegenerative disorders such as SCA3 and HSP [8, 13]; however, while tibial nerve CSA was additionally decreased in SCA3 and HSP, no such change in CSA was observed in our FRDA cohort. The existence of an unchanged CSA of lower extremity nerves also distinguishes FRDA from other sensory neuronopathies such as the cerebellar ataxia, neuropathy and vestibular areflexia syndrome (CANVAS), where degeneration of dorsal root ganglia leads to CSA reduction in peripheral nerves [24]. Our findings are in line with sonographic studies conducted in FRDA which found no change in lower extremity nerve CSA compared to healthy controls while observing an increase in CSA in upper extremity nerves [7, 25]. The authors of those sonography studies have hypothesized that a dorsal root ganglionopathy, leading to secondary atrophy of peripheral nerves, reflects the predominant underlying pathomechanism for their observation. Further support comes from neuropathologic studies that previously reported on a more pronounced atrophy of the gracile fasciculus (lower extremities) compared to the cuneate fasciculus (upper extremities) within the dorsal column of the spinal cord [7, 26, 27]. While these findings would explain an atrophy of peripheral nerves, the unchanged lower extremity nerve CSA in our FRDA cohort as well as in the reported sonographic studies favor a more complex pathophysiologic mechanism compared to pure sensory neuronopathies or dorsal root ganglionopathies. Even though our study focused on the characterization of the tibial nerve as an example for a mixed sensory‐motor nerve, it is important to consider that mixed nerves contain mostly sensory fibers [28].

In our cohort, changes of tibial nerve *ρ* and T2_app_ at the thigh and lower leg inversely correlated with the SARA sum score as a measure of disease severity as well as with disease duration. At the thigh, both tibial nerve *ρ* and T2_app_ additionally correlated well with sural SNAPs, while tibial nerve T2_app_ also correlated with tibial nerve CMAPs. The correlations observed between MRN markers and clinical findings, as well as NCS parameters, are particularly important as they support the assumption that changes in *ρ* and T2_app_ reflect pathophysiologically relevant microstructural changes in respective nerves. This underscores the potential of *ρ* and T2_app_ as beneficial quantitative and objective imaging biomarkers that may aid in estimating disease progression and treatment effects in FRDA patients without being influenced by subjective factors such as fluctuations in a patient's overall health status or clinician bias. Furthermore, T2 relaxometry markers have previously proven to detect nerve lesions prior to clinical and electrophysiologic symptom manifestation, e.g. in hereditary transthyretin amyloidosis [11, 15, 19]. This might make them even more sensitive to detect early disease progression or treatment response than gold standard clinical scores and NCS. However, longitudinal follow‐up studies are needed to support this hypothesis.

The cross‐sectional design of our study limits any assessment of the temporal evolution and progression of structural peripheral nerve damage in FRDA based on quantitative MRN markers, making future longitudinal MRN studies essential to evaluate the potential of imaging biomarkers to reliably monitor the natural course of the disease, predict outcomes, or measure response to treatment in clinical trials once first therapeutic options emerge. Another limitation is the small number of participants in our study cohort. However, with a prevalence of 1 in 50,000 people in the US, FRDA is considered a rare disease. In addition, FRDA patients with a co‐diagnosis of diabetes, occurring in approximately 8.7% of all FRDA patients [29], were excluded from study participation as diabetes would be a competing cause for peripheral neuropathy development.

Our study provides an advanced characterization of peripheral nerve involvement in FRDA that might help to understand the mechanisms involved in the multisystemic disease evolution in FRDA. The observed good correlation between the microstructural quantitative MRN parameters tibial nerve *ρ* and T2_app_ and disease duration, the SARA sum score, as well as NCS results might make them promising objective imaging biomarkers to monitor disease progression and therapeutic responses on a microstructural level in potential future clinical trials.

## Author Contributions

**Heike Jacobi:** conceptualization, writing – original draft, project administration, methodology, data curation, formal analysis, supervision, investigation, validation. **Markus Weiler:** writing – review and editing, data curation, formal analysis, supervision, investigation, validation, software. **Georges Sam:** writing – review and editing, data curation, formal analysis, investigation. **Sabine Heiland:** writing – review and editing, data curation, formal analysis, supervision, investigation, validation. **John M. Hayes:** writing – review and editing, data curation, formal analysis, supervision, visualization. **Martin Bendszus:** writing – review and editing, data curation, formal analysis, supervision. **Wolfgang Wick:** writing – review and editing, data curation, formal analysis, supervision. **Jennifer C. Hayes:** conceptualization, writing – original draft, project administration, methodology, visualization, data curation, formal analysis, supervision, investigation, validation, software.

## Disclosure

H.J. received the Olympia Morata stipend grant from the Medical Faculty of the University of Heidelberg. M.W. reports no disclosures related to the present work. S.H. received research support from the German Research Foundation (SFB 1118). J.M.H. reports no disclosures. M.B. received research support from the German Research Foundation (SFB 11158). W.W. received research support from Apogenix, Pfizer, and Roche outside the topic of this article. He provided expertise or consulting to Enterome, MSD, Roche, and Servier independent of the topic of the work presented here. J.C.H. reports no disclosures related to the present work.

## Conflicts of Interest

The authors declare no conflicts of interest.

## Data Availability

The data that support the findings of this study are available on request from the corresponding author. The data are not publicly available due to privacy or ethical restrictions.
